# Berberine supplementation modulates the somatotropic axis and ameliorates glucose tolerance in dairy goats during late gestation and early lactation

**DOI:** 10.1186/s12917-022-03452-9

**Published:** 2022-09-24

**Authors:** Navid Ghavipanje, Mohammad Hasan Fathi Nasri, Seyyed Homayoun Farhangfar, Seyyed Ehsan Ghiasi, Einar Vargas-Bello-Pérez

**Affiliations:** 1 Department of Animal Science, Faculty of Agriculture, University of Birjand, Birjand, 97175-331 Iran; 2 Department of Animal Sciences, School of Agriculture, Policy and Development, University of Reading, P.O. Box 237, Earley Gate, Reading, RG6 6EU UK

**Keywords:** Berberine, Glucose tolerance test, Somatotropic axis, Saanen goat, Transition period

## Abstract

**Background:**

Pregnancy, parturition, and the onset of lactation represent an enormous physiological and hormonal challenge to the homeostasis of dairy animals, being a risk for their health and reproduction. Thus, as a part of the homothetic changes in preparturition period, goats undergo a period of IR as well as uncoupled GH/IGF-1 axis. The objective for this study was to determine the effect of berberine (BBR) during the peripartal period on hormonal alteration and somatotropic axis in dairy goats as well as glucose and insulin kinetics during an intravenous glucose tolerance test (IVGTT). At 21 days before the expected kidding date, 24 primiparous Saanen goats were assigned randomly to 4 dietary treatments. Goats were fed a basal diet from wk. 3 antepartum (AP) until wk. 3 postpartum (PP) supplemented with 0 (CTRL), 1 (BBR1), 2 (BBR2), and 4 (BBR4) g/d BBR. Blood samples were collected on days − 21, − 14, − 7, 0, 7, 14, and 21 relative to the expected kidding date. An IVGTT was also performed on day 22 PP.

**Results:**

Compared with CTRL, supplementation with either BBR2 or BBR4 increased DMI at kidding day and PP, as well as body conditional score (BCS) and milk production (*p* ≤ 0.05). On d 7 and 14 PP plasma glucose was higher in BBR2- and BBR4-treated than in CTRL. The glucagon concentration was not affected by BBR during the experimental period. However, supplemental BBR indicated a tendency to decrease in cortisol concentration on days 7 (*p* = 0.093) and 14 (*p* = 0.100) PP. Lower plasma GH was observed in BBR than in non-BBR goats (*p* ≤ 0.05). Plasma IGF-1 concentration was enhanced in both BBR2 and BBR4 at kidding and day 7 PP (*p* ≤ 0.05). During the IVGTT, glucose area under the curve (AUC), clearance rate (CR), T_1/2_, and T_basal_ was lower (*p* ≤ 0.05) in both BBR2 and BBR4 goats as compared with CTRL. Likewise, the insulin CR was higher (*p* ≤ 0.05) in goats receiving either BBR2 or BBR4 which was accompanied by a lower insulin T_1/2_ and AUC.

**Conclusions:**

Altogether, our results indicated an improved glucose and insulin status along with the modulation of the somatotropic axis and glucose and insulin response to IVGTT in dairy goats supplemented with 2 and 4 g/d BBR.

## Background

The period from late gestation to early lactation, whether in dairy goats [1] or dairy cows [2] consists of a complex interplay of multiple pathways, including metabolic and hormonal adaptations. These changes are related to the partitioning of the nutrient supply for milk production and occur in a chain reaction fashion that begins within 3 weeks before calving and lasts for three to 4 weeks after parturition [2, 3]. The regulation of nutrient partitioning is arranged by complex interactions between hormones [i.e., insulin, growth hormone (GH), Insulin-like growth factor 1 (IGF-1), and glucocorticoids] aiming to favor glucose supply for milk synthesis that involves insulin action and the somatotropic axis [4]. During early lactation, suppressed insulin sensitivity along with uncoupled GH/IGF-1 axis stimulates the mobilization of body fat reserves [4, 5]. The uncoupling of the somatotropic axis is marked by lower expression of growth hormone receptor (GHR) in the liver and consequently decreased IGF-1 synthesis in the liver [6]. These results in reduced negative feedback of IGF-1 on growth hormone (GH) secretion, and, consequently, increased GH and reduced IGF-1 concentrations [6, 7]. As lactation progresses, the GH/IGF-1 axis becomes recoupled that led to an increase in IGF-1 concentrations [8].

Increased GH concentrations during early postpartum (PP) act as an antagonistic to insulin by enhancing lipolysis and developing insulin resistance (IR) to help direct nutrients from insulin-sensitive tissues to the lactating mammary gland [5, 9]. Thus, as a part of the homothetic changes in preparturition period, goats [1] undergo a period of IR, similar to cows [5]. It is well established that PP cows often exhibit symptoms similar to type 2 diabetes (T2DM), including elevated plasma free fatty acid concentrations and decreased sensitivity of tissue to the presence of insulin which plays a major role in the development of many metabolic disorders [10]. It is well established that transition period in dairy goats [11] is associated with reduced insulin and elevated GH concentrations like cows [6, 12], intensified mobilization of body fats [12], and a greater degree of IR (5). Similar to observations in dairy cows, a recent paper demonstrated that transition goats have lower insulin and glucose as well as higher GH levels [1]. However, in contrast to the comprehensive information on endocrine regulation of nutrient partitioning in dairy cows [5, 13], little is known about endocrine and metabolic mechanisms in early-lactation goats.

Berberine (BBR), a naturally occurring protoberberine alkaloid, is a pharmacological component isolated from certain species of flowering plants such as *Berberidaccae*, *Coptis rhizomes,* and *Hydrastis Canadensis* [14]. BBR-containing plants have historically been used as an antimicrobial agent in the treatment of infective diarrhea [15]. However, synthetic BBR has an intense yellow powder, odor less with a characteristic alkaloidal bitter taste [14]. Recent studies showed that BBR is a promising active component with potential to produce several bioactive derivatives [15]. BBR has significant anti-hypertensive, anti-arrhythmic, anti-hyperglycemic, anti-cancer, anti-depressant, neuro-protective, anti-oxidant, anti-inflammatory, hypolipidemic activity [14]. In recent years, the focus of BBR research has shifted towards potential therapeutic benefits in treating metabolic dysfunctions, such as T2DM, with data indicating glucose- and lipid-lowering effects [14, 16]. It has been suggested that BBR may overcome IR and regulating signaling pathways, such as the AMPK and JNK pathways [17].

Taken together, the aim of the present study was to determine the effect of BBR supplementation on glucose metabolism and somatotropic axis in dairy goats during late gestation and early lactation. Previous findings within this project confirmed that the BBR-fed does showed improvement in the energy balance (EB) around preparturition period [18]. Therefore, the hypothesis of this study was that BBR ingestion during the transition from late pregnancy to early lactation, affects glucose metabolism and IR as well as the somatotropic axis.

## Materials and methods

### Animals, husbandry, feeding, and BBR supplementation

The experimental protocol and implemented procedures were reviewed by the Animal Welfare and Ethical Review Board of the Department of Animal Science, University of Birjand under the approved ID project 5506 and were conducted in accordance with ARRIVE [19] guidelines and regulations. This experiment was performed at the experimental farm of the Faculty of Agriculture, University of Birjand, Iran (longitude and latitude, 37.42° N and 57.31° E, 1491 m above sea level, and an annual average rainfall of 171 mm) from August 2019 to December 2019.

Twenty-four primiparous Saanen dairy goats [375 ± 23 days of age; 45 ± 3.5 kg body weight (BW); 3.0 ± 0.5 body condition score (BCS), mean ± SD] were collected from experimental farm of the Faculty of Agricultural Research Station, University of Birjand, Iran and were housed in individual stalls (1.8 m × 1.6 m) in a ventilated enclosed barn from 50 days before their expected kidding date through 21 days PP. The first 29 days were used for adaptation and the remaining 42 days were used for measurements. The estrus synchronization was performed using a progesterone-releasing intravaginal device (0.3 g of progesterone; Pfizer Animal Health, West Ryde, New Zealand) which was removed after 19 days of insertion followed by intramuscular injection with 500 international units (IU) of eCG (Syncropart; CEVA Santé Animale, Libourne, France). Antepartum (AP) and post-partum (PP) basal diets were formulated to be isocaloric (2.60 and 2.90 Mcal ME dry matter basis in AP and PP diets, respectively) and isonitrogenous (18.5 and 15.5% CP dry matter basis in AP and PP diets, respectively) and to meet NRC [20] nutritional requirements of each period. Goats were fed (allowing for 5 to 10% refusal) ad libitum total mixed rations twice daily at 06:00 and 16:00 h (Table 1) and had free access to water during the experimental period. Pure Berberine HCL powder (BBR) was purchased from Bulk Supplement Factory (Bulk Supplements, Eastgate, Henderson, USA). This product had no other ingredients. BBR was supplemented at a rate of 0 (CTRL), 1 (BBR1), 2 (BBR2), and 4 (BBR4) g/d per doe, which corresponded to 0, 25, 50, and 100 mg/kg BW, respectively. To ensure full BBR consumption, it was encapsulated in gelatin capsules (Irancapsul, Tehran, Iran). All BBR capsules were labelled according to each treatment and were orally administrated to each doe before morning feeding with a balling gun (Pars Khavar, Tehran, Iran); while the CTRL group received empty capsules. Due to the lack of comparable data regarding the effects of BBR in ruminants, these pharmacological doses were chosen based on the observed effects of BBR on metabolic dysfunctions in non-ruminant species [16, 22] and humans [23]. Of note, all samplings were performed in a blinded manner, meaning that persons that fed animals and decided the random assignment of the goats were different.

**Table 1:** Ingredients and nutrient composition (DM basis) of pre- and post-partum diets

	Diets	
	Pre-partum ^a^	Post-partum ^b^
Ingredient (% of DM)		
Alfalfa hay	4.00	29.5
Corn silage	34.3	10.8
Wheat straw	17.9	5.00
Barley grain, ground	7.70	10.8
Corn grain, ground	31.5	22.2
Soybean meal	1.00	17.0
Wheat bran	1.80	2.20
Calcium–carbonate	0.90	1.00
Minerals and vitamins premix ^c^	0.90	0.50
Salt	0.00	1.00
Chemical composition		
ME, Mcal/kg of DM ^d^	2.60	2.90
Crude protein (% DM)	18.5	15.5
Ether extract (% DM)	2.50	2.50
Ash (% DM)	7.60	8.00
Neutral detergent fiber (% DM)	43.0	37.3
Non-fibrous carbohydrates (% DM) ^e^	38.0	36.7

^a^From 50 days before parturition until kidding

^b^From kidding until 21 days of lactation

^c^Containing vitamin A (250,000 IU/kg), vitamin D (50,000 IU/kg), vitamin E (1,500 IU/kg), manganese (2.25 g/kg), calcium (120 g/kg), zinc (7.7 g/kg), phosphorus (20 g/kg), magnesium (20.5 g/kg), sodium (186 g/kg), iron (1.25 g/kg), sulfur (3 g/kg), copper (1.25 g/kg), cobalt (14 mg/kg), iodine (56 mg/kg) and selenium (10 mg/kg)

^d^Cornell Net Carbohydrate and Protein System predicted based upon measured feedstuff nutrient composition and actual DMI using SRNS [21]

^e^Non-fibrous carbohydrates (NFC) were estimated according to the equation: NFC = 100 − (NDF + CP + EE + Ash) [20]

### Measurements and laboratory analysis

The amounts of feed offered and refused were recorded daily throughout the experiment. Samples of TMR and orts were separately pooled and ground in a hammer mill with a 1-mm screen (Arthur Hill Thomas Co., Philadelphia, PA). Dry matter (DM; method no. 930.15), crude protein (CP; method no. 990.03), ether extract (EE; method no. 945.16) and ash (method no. 967.05) were measured (three replicates) according to the procedures of AOAC [24]. Neutral detergent fiber (NDF) was measured (Fibertec 1010, Tecator, Sweden) as described by Van Soest et al. [25]. All goats were weighed using a calibrated scale (ASA2200, Sepahan Towzin Co., Isfahan, Iran) on days − 21, − 14, − 7, 0, 7, 14, and 21 relative to kidding and at the same time Body condition score (BCS) was taken by the same individual as described by Villaquiran et al. [26].

After parturition, all goats were milked twice per day at 05:30 and 15:30 using a portable milking machine (Tim Gibson Ltd., Bedale, UK). Milk yield was recorded at each milking for the duration of the experiment. Pre-prandial blood samples (10 ml/goat) were collected immediately after morning milking and before feeding, from the jugular vein of each doe using Li-heparin containing tubes (RotexMedica, Germany) on days − 21, − 14, − 7, 0, 7, 14, and 21 relative to kidding. The tubes were immediately placed on ice and instantly transported to the laboratory within 30 minutes. Blood samples were centrifuged at 3000×g for 15 min; plasma was obtained by centrifuging it was then stored (− 20 °C) until analysis.

Plasma glucose was detected using commercial kits (Pars Azmun Co. Ltd., Tehran, Iran) by an autoanalyzer (BT 1500, Biotecnica SpA, Rome, Italy). Plasma insulin (Insulin AccuBind®, Kit number: 2425-300B, Monobind Inc., CA, USA), glucagon (Kit number: 138030, MyBioSource, Inc., CA, USA), and cortisol (Kit number: CSB-E18048G, Cusabio Inc., Houston, USA) concentrations were determined using the commercially available goat enzymelinked immunosorbent assay kits, according to the manufacturer’s instruction. The standard curves were prepared at concentrations 7.5 to 240 IU/l for insulin and 100 to 3200 ng/l for glucagon. The sensitivity of these methods was 0.27 for insulin, 5.24 for glucagon, and 5.05 for glucagon. Intra- and inter-assay coefficients of variation for insulin, glucagon, and cortisol were below 8, 10, and 9% respectively. Plasma GH and IGF-1 were measured by radioimmunoassay (RIA) as described previously [27]. Intra- and inter-assay coefficients of variation for GH and IGF-I RIA were below 9 and 11%, respectively. All samples were analyzed in duplicate.

### Intravenous challenge

An intravenous glucose challenge was performed on day 22 PP to examine the insulin responsiveness to glucose load, following a method from Salin et al. [25]. Three goats were randomly selected from each treatment group according to their body weight. The goats were fitted with sterile indwelling jugular catheters (14G × 5.1 cm; Jelco™, Johnson and Johnson, Mumbai, India) on the day prior to the IVGTT. After an overnight fast the pre-challenge blood samples (times − 15, − 10, and − 5 min) were collected to define the basal concentration of the metabolites following which an intravenous bolus dose of glucose (500 mg of glucose/kg of body weight as sterile 50% solution, Zoopha®, Parnian CO., Iran) was administered at room temperature within a period of 30s. Subsequently, blood sampling was done at 5, 10, 15, 20, 30, 45, 60, 90, 120, and 180 min after injection using heparinized vacuum tubes. Plasma was centrifuged at 3000×g for 15 min and then stored (− 20 °C) until analysis of glucose and insulin. After measuring blood metabolites, the corresponding data were evaluated in SAS 9.2 software. The NLIN procedure was used to fit exponential curves for glucose concentration during the metabolic challenge using the following equation [28]:

$$F\ (t)=A\times {e}^{\left(-k\times t\right)}$$

Where F(t) is the metabolite concentration at time t; A is the maximum value of metabolite; t is the time (min); and k is the regression coefficient. Both A and K estimations were calculated. The following parameters were calculated based on the fitted data. The clearance rate (CR) of glucose and insulin was calculated by using the following formula [28]:

$$\mathrm{Clearance}\ \mathrm{rate}\ \left(\mathrm{CR};\%\min \right)=\left\{\left(\ln \left[\mathrm{ta}\right]-\ln \left[\mathrm{tb}\right]\right)/\left(\mathrm{tb}-\mathrm{ta}\right)\right\}\times 100$$

Plasma half-life (T _1/2_) of glucose and insulin or the time to reach half-maximal concentrations calculated using the following formula [28]:

$${\mathrm{T}}_{1/2}\ \left(\min \right)=\left\{\left[\ln (2)\right]/\mathrm{CR}\right\}\times 100$$

T _basal_ or the time to reach basal glucose and insulin concentration was calculated as follows [28]:

$${\mathrm{T}}_{\mathrm{basal}}\left(\min \right)=\left(\left(\mathrm{Ln}\left(\mathrm{ta}\right)-\mathrm{Ln}\left(\mathrm{tb}\right)\right)/\mathrm{CR}\right)\times 100$$

Where [ta] is the concentration of metabolite at time a (ta), and [tb] is the concentration of metabolite at time b (tb).

The areas under the curve (AUC) of glucose and insulin during IVGTT were calculated after drawing the curve for glucose and insulin using the trapezoidal method [28].

### Statistical analyses

All data were analyzed using the MIXED procedure of SAS version 9.2 (SAS/STAT, SAS Institute Inc., Cary, NC). The REPEATED statement of SAS was used for dependent variables measured over time. The model included the fixed effects of BBR (levels: 0, 1, 2, and 4 g/d), time of measurement (level: day relative to kidding), and their interaction. Each variable analyzed was subjected to three covariance structures including autoregressive order (AR1), spatial power (SP), and unstructured (UN) and the one resulting in the lowest Akaike information criterion was chosen. The results are presented as least squares means (LSM) ± standard error. The differences in LSM were tested using the Tukey-Kramer procedure. For all data, significance was declared at *p* ≤ 0.05, and tendency was declared at 0.05 < *p* ≤ 0.10.

## Results

### Animal performance

During the AP period, goats fed BBR2 and BBR4 treatments tended (*p* = 0.100) to have greater DMI than goats fed BBR1 and CTRL (Table 2). Likewise, supplementation of BBR2 and BBR4 increased DMI during the kidding day (*p* = 0.016) and PP period (*p* = 0.021). Moreover, intake of metabolizable protein (MP) and net energy for lactation (NEL) were higher (*p* ≤ 0.05) for goats receiving supplemental BBR2 or BBR4 at AP, kidding day, and PP period. No differences (*p* > 0.05) between CTRL and BBR supplemented groups were observed in goat’s BW at AP and PP as well as on kidding day. In addition, none of the BBR supplemented diets influenced (*p* > 0.05) BCS at AP; while BCS was increased in goats fed BBR2 and BBR4 at kidding day and PP period. Both BBR2 and BBR4 increased (*p* = 0.007) milk yield. Compared to CTRL and BBR treatments, BBR2 led to higher milk production and production efficiency in PP goats.

**Table 2:** Effect of BBR supplementing on ante-partum, kidding day and post-partum performance of transition dairy Saanen goats

Item^1^	Treatments ^2^				SEM^4^	*P*-value
	CTRL	BBR1	BBR2	BBR4		
Ante-partum (AP)						
DMI (kg/d)	1.58	1.57	1.61	1.63	0.021	0.100
MP intake (g/d)	115.7^b^	116.1^b^	135.1^a^	135.9^a^	4.655	0.091
NEL intake (Mcal/d)	1.53^b^	1.54^b^	1.78^a^	1.78^a^	0.069	0.027
Mean BW (kg)	50.3	51.2	51.0	50.3	1.60	0.892
Mean BCS	3.40	3.62	3.70	3.60	0.085	0.120
Kidding day						
DMI (kg/d)	1.08^b^	1.11^b^	1.33^a^	1.38^a^	0.054	0.016
MP intake (g/d)	89.29^b^	91.60^ab^	110.4^a^	113.1^a^	5.287	0.020
NEL intake (Mcal/d)	1.14^b^	1.16^b^	1.40^a^	1.43^a^	0.067	0.013
Mean BW (kg)	42.9	44.5	45.5	44.2	1.52	0.701
Mean BCS	3.05^b^	3.45^ab^	3.90^a^	3.95^a^	0.165	0.003
Post-partum (PP)						
DMI (kg/d)	2.21^b^	2.28^b^	2.47^a^	2.42^a^	0.058	0.021
MP intake (g/d)	340.3^b^	375.6^ab^	443.7^a^	428.4^a^	20.257	0.030
NEL intake (Mcal/d)	2.75^b^	3.03^ab^	3.58^a^	3.46^a^	0.163	0.013
Mean BW (kg)	43.0	43.8	45.5	44.5	0.755	0.680
Mean BCS	3.07^b^	3.10^ab^	3.41^a^	3.20^a^	0.068	0.010
Milk yield (kg/d)	1.66^b^	1.96^b^	2.48^a^	2.10^ab^	0.143	0.007
Production efficiency ^3^	0.833^b^	1.01^b^	1.18^a^	1.00^b^	0.070	0.019

Values are least-square means

^a–b^Means with different superscript letters within a row are different (*P* ≤ 0.05); the highest value are indicated with letter a, followed by b and c

^1^AP data cover the last 21 d of gestation and PP data cover the first 21 d of lactation

^2^CTRL, BBR1, BBR2 and BBR4 Supplemented with 0, 1, 2 and 4 g BBR/d, respectively

^3^Production efficiency = average daily milk yield (kg/d)/average daily DMI (kg/d)

^4^Pooled standard error of mean

### Glucose metabolism and related hormones

**Fig. 1:**
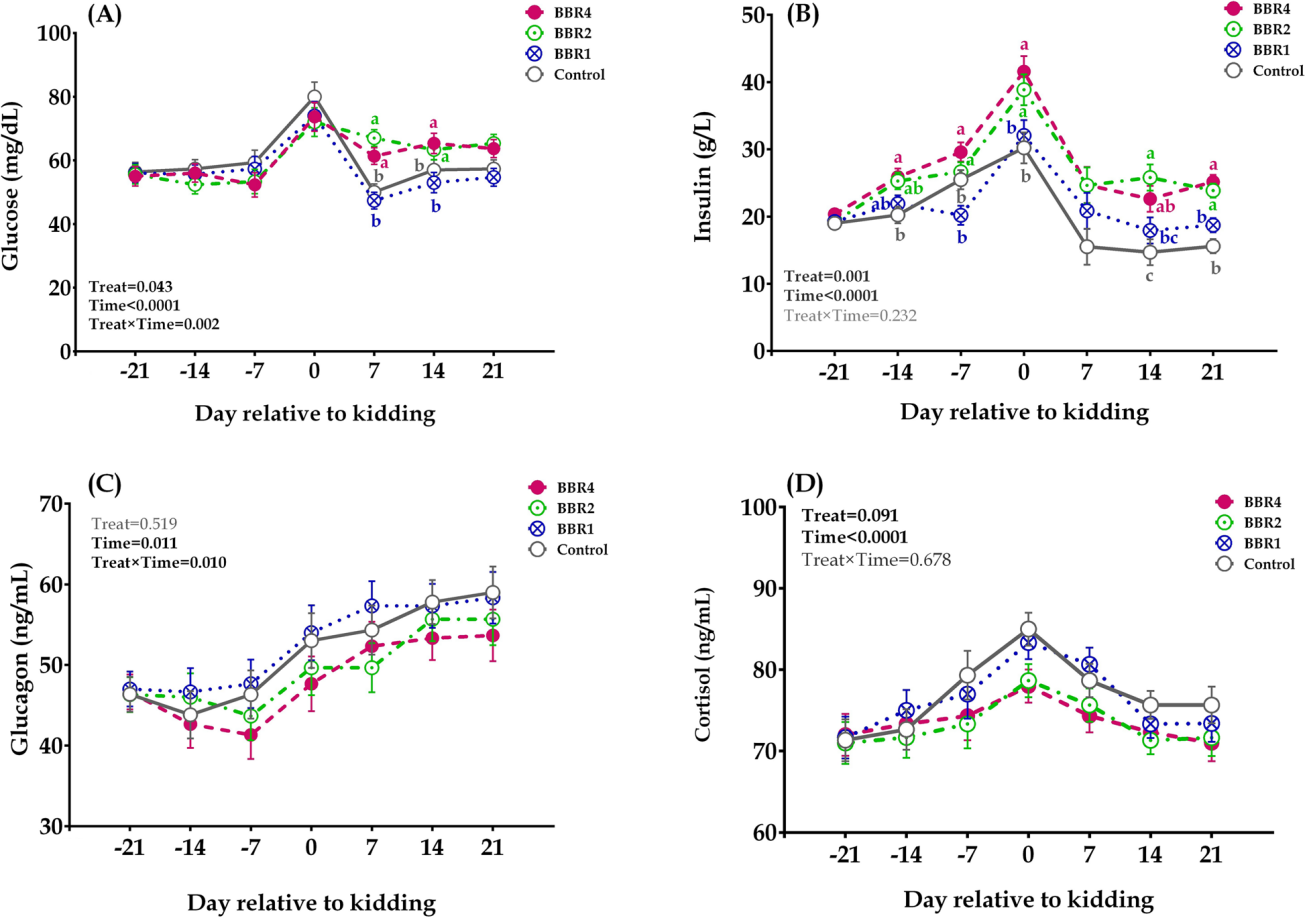
Concentrations of plasma glucose (**A**), insulin (**B**), glucagon (**C**) and cortisol (**D**), in does supplemented with 0 (CTRL), 1 (BBR1), 2 (BBR2), and 4 (BBR4) g/d of BBR from day 21 AP until day 21 PP. Data are presented as LSM ± SE; LSM with different letters (a, c) differ (*p* ≤ 0.05) at the respective time point

Plasma glucose concentration (Fig. 1A) peaked on kidding day, dropped down immediately after parturition, and slightly increased thereafter (*p* < 0.001). In addition, plasma glucose indicated a BBR effect (*p* = 0.043) as well as the interactions between BBR and time (*p* = 0.002). During AP period there were no significant BBR effects on plasma glucose, however, after parturition on days 7 (*p* = 0.002) and 14 (*p* = 0.042) glucose concentration was higher in BBR2 and BBR4 than in CTRL. Plasma insulin concentration (Fig. 1B) increased (*p* < 0.001) as kidding approached and decreased markedly after kidding in all groups. Plasma insulin was higher (*p* = 0.001) in BBR-treated than non-BBR goats, but indicated no further treatment × time differences (*p* = 0.232). On days − 14, − 7, 0, 14, and 21 relative to parturition, insulin concentration was higher (*p* ≤ 0.05) in BBR2 and BBR4 compare to CTRL. There were no significant effects between the treatment groups for glucagon concentration (Fig. 1C); however, both BBR2 and BBR4 showed numerically lower plasma glucagon throughout the transition period. Plasma cortisol (Fig. 1D) varied during the transition period with peaks at kidding (*p* < 0.001). In addition, BBR ingestion indicated a tendency to decrease in cortisol concentration (*p* = 0.09) without a noticeable treatment × time effect (interaction *p* = 0.676). Of note, plasma cortisol tended to decrease with increasing BBR supplementation on days 7 (*p* = 0.093) and 14 (*p* = 0.100) PP.

### Somatotropic Axis in blood plasma

**Fig. 2:**
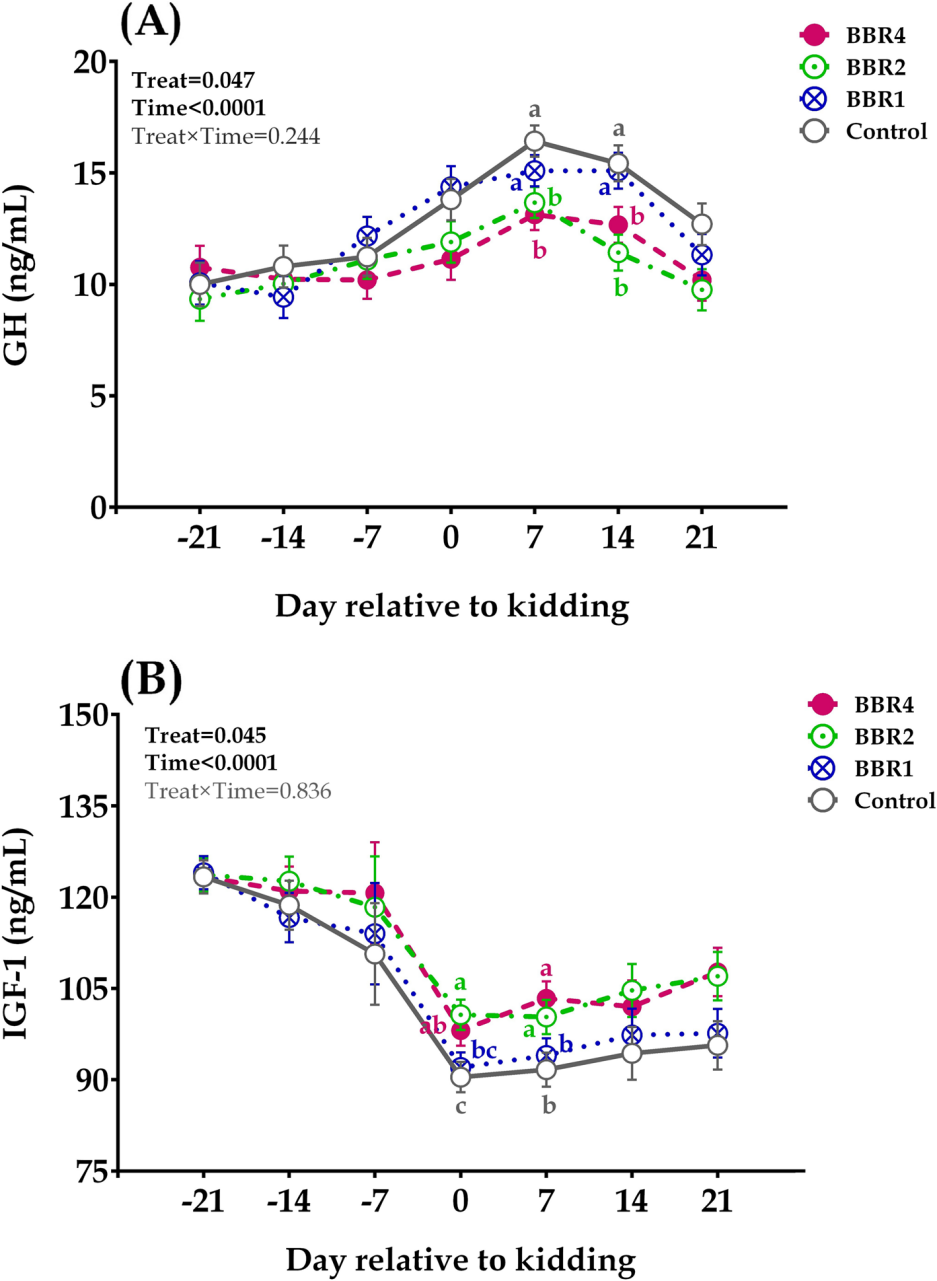
Concentrations of plasma growth hormone (GH, **A**) and insulin growth factor-1 (IGF-1, **B**) in does supplemented with 0 (CTRL), 1 (BBR1), 2 (BBR2), and 4 (BBR4) g/d of BBR from day 21 AP until day 21 PP. Data are presented as LSM ± SE; LSM with different letters (a, c) differ (*p* ≤ 0.05) at the respective time point

The GH concentration in blood plasma (Fig. 2A) progressively increased during AP period and early lactation, which peaked on day 7 after kidding and slightly decreased (*p* < 0.001) thereafter. Overall, lower (*p* = 0.047) plasma GH was observed in BBR than in non-BBR goats. At AP period, GH concentration did not differ between treatment groups (*p* ≤ 0.05). However, at kidding day, the BBR supplement tended (*p* = 0.095) to decrease plasma GH. Likewise, on days 7 (*p* = 0.006) and 14 (*p* = 0.002) PP, BBR2- and BBR4-supplemented does had a lower plasma GH concentration.

Plasma IGF-I concentration (Fig. 2B) was highest on d 21 AP and decreased (*p* < 0.001) in all groups as kidding approached. Overall, BBR increased (*p* = 0.045) plasma IGF-I and the interactions between BBR supplements and time were not significant (*p* = 0.838). During the AP period (days − 21, − 14, and − 7) no significant effect was found among BBR-treated than in non-BBR goats for IGF-1. However, both BBR2 and BBR4 groups increased the plasma IGF-1 concentration at days 0 (*p* = 0.050) and 7 (*p* = 0.041) relative to kidding. Moreover, a tendency to increase (*p* = 0.100) was observed with BBR supplementation on day 21 PP.

### Glucose and insulin responses during IVGTT

**Fig. 3:**
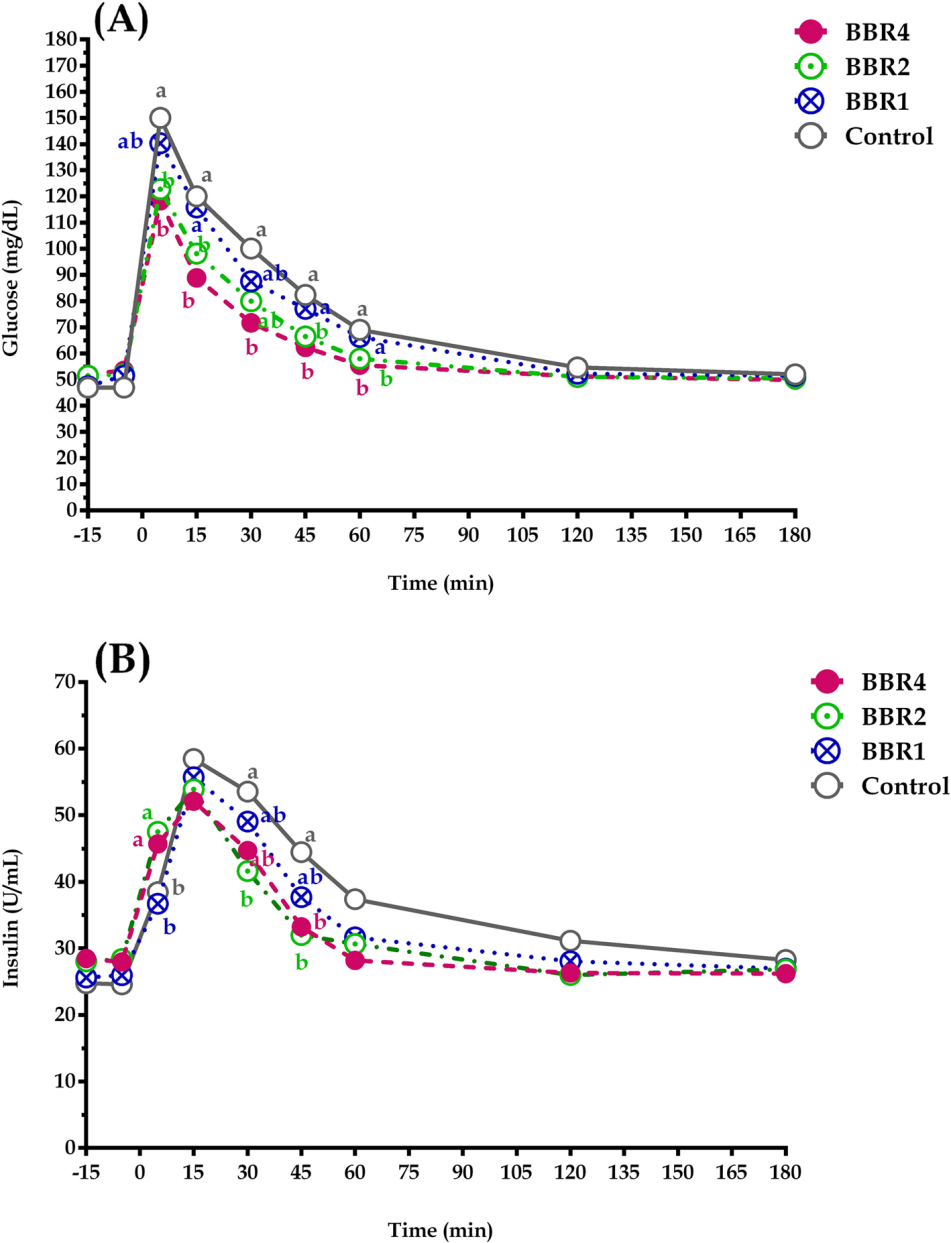
Responses in (**A**) plasma glucose, (**b**) plasma insulin concentrations to an glucose infusion (0.500 mg/kg of BW) at time zero in does supplemented with 0 (CTRL), 2 (BBR2), and 4 (BBR4) g/d of BBR from day 21 AP until day 21 PP. Data are presented as LSM ± SE; LSM with different letters (a, b) differ (*p* ≤ 0.05) at the respective time point

Glucose and insulin responses during IVGTT are shown in Table 3 and Fig. 3. Basal plasma glucose concentration was not affected (*p* = 0.417) by BBR treatments. Plasma glucose increased sharply and then gradually decreased following glucose infusion. The peak of plasma glucose concentration was reached within 15 min after the infusion and was lower (*p* = 0.022) in BBR2 and BBR4 does. Glucose AUC (*p* = 0.031), T _½_ (*p* = 0.020), and T_basal_ (*p* = 0.001) was lower in both BBR2 and BBR4 goats compared with CTRL. Glucose clearance rate (CR) was higher (*p* = 0.006) with increasing BBR supplementation. Plasma insulin also increased following the intravenous glucose infusion. Dietary BBR2 and BBR4 supplementation enhanced basal insulin concentration during the IVGTT (*p* = 0.029). The insulin clearance rate was higher (*p* = 0.013) in goats receiving either BBR2 or BBR4, this was accompanied by a lower insulin T_1/2_ (*p* = 0.023) and AUC (*p* = 0.001) in these groups.

**Table 3:** Effects of BBR supplementing on glucose and insulin kinetics of transition dairy Saanen goats during intravenous glucose tolerance test (IVGTT)

Item^1^	Treatments ^2^				SEM^3^	*P*-value
	CTRL	BBR1	BBR2	BBR4		
Glucose						
Basal glucose (mg/dl)	49.5	47.2	51.0	52.7	2.33	0.417
CR (% min)	1.59^c^	1.70^bc^	1.88^ab^	1.95^a^	0.031	0.006
T _½_ (min)	43.80^a^	41.3^ab^	36.8^bc^	35.9^c^	0.940	0.020
T _basal_ (min)	70.14^a^	63.82^a^	52.90^b^	50.85^b^	1.32	0.001
AUC 120	1376^a^	1334^a^	1206^b^	1195^b^	12.94	0.031
Insulin						
Basal insulin (mg/dl)	24.7^b^	25.8^b^	28.2^a^	28.3^a^	0.593	0.029
CR (% min)	1.53^c^	1.55^bc^	1.89^ab^	1.99^a^	0.061	0.013
T _½_ (min)	56.8^a^	54.4^ab^	46.5^bc^	44.6^c^	1.46	0.023
AUC 120	710^a^	689^a^	622^b^	617^b^	11.85	0.001

Values are least-square means

^a–c^Means with different superscript letters within a row are different (*P* ≤ 0.05); the highest value are indicated with letter a, followed by b and c

^1^CR; The clearance rate of glucose and insulin after their maximum concentrations as a consequence of glucose injection, T _½_; the half-life or the time needed for glucose and insulin levels to return to half of their maximum concentrations. T _basal_; The time needed for glucose and insulin to return to their basal levels. AUC; The area under the curve glucose and insulin after glucose injection (calculated using trapezoid method)

^2^CTRL, BBR1, BBR2 and BBR4 supplemented with 0, 1, 2 and 4 g BBR/d, respectively

^3^Pooled standard error of mean

## Discussion

In dairy goats, reducing the effectiveness of insulin and metabolic alteration at the onset of lactation seems [1, 29, 30] to facilitate the supply of available glucose to the mammary gland to be used as energy for milk synthesis [1, 31]. However, contrary to the extensively studied metabolic adaptation of transition dairy cows [5, 6, 13], endocrine changes, insulin responsiveness, and uncoupled GH-IGF-1 axis during late gestation and early lactation remains under investigation in dairy goats. In this regard, some researchers have investigated changes in insulin response of dairy goats with regard to physiological state [31], genetic merit for milk yield [32], and energy intake [33]. Although, it has been recently reported the differences in metabolic responses of dairy goats according to the level of milk production as well as the mechanisms of nutrient partitioning [1]. In a companion study, we observed that BBR supplementation led to reduction in body fat reserve mobilization as indicated by the lower NEFA and BHBA circulation as well as enhanced energy balance (EB) with faster recovery during the first 3 wk. PP [18]. Therefore, the central aim of present study was to clarify whether the favorable EB of BBR-supplemented dairy goats during transition state was related to enhancement of the regulation in somatotropic axis and glucose tolerance.

### Animal performance

During late gestation and early lactation, insulin resistance is probably followed by negative energy balance (NEB) and is characterized by increase IR [4], which along with some other factors leads to decrease in DMI [34]. The levels of DMI in CTRL and BBR supplemented goats were consistent with the predicted values previously published [29]. We observed no difference in DMI during the AP period in response to BBR supplementation; however, improved DMI was evident at kidding and PP in goats consumed BBR2 and BBR4 treatments. In addition, we found that the intake of NEL and MP was also higher in goats fed BBR2 and BBR4. It has been reported [5] that hormone-induced adaptations in transition cows lead to reductions of DMI and serious imbalances of energy status. In addition, a positive correlation between DMI and intake of NEL and MP, as well as EB, has been reported in goats [1] and cows [3]. Both the BW and BCS were in line with the findings of Zamuner et al. [1] regards transition goat profiles. In other animal models such as in rats, it has been reported that oral administration of 50 and 100 mg BBR/kg BW (doses similar to the present study) decreased IR and enhanced glucose metabolism [22]. Shi et al. [35] reported that BBR regulates the insulin-signaling pathway, and it can increase the sensitivity of insulin receptor, and thereby reduces IR. In addition, shinjo et al. [16] reported that BBR activates AMPK, which plays a key role in regulation of whole-body energy homeostasis. In this regard, it well known that AMPK activation may enhance insulin sensitivity [36]. Our findings agree with Smith et al. [37] and Yousefi et al. [38] who reported that when attenuating IR in transition cows, positive effects on NEB can be observed and thereupon on DMI and milk yield. The increased milk production in goats supplemented with BBR2 and BBR4 is likely due to a decline in NEB and an increase in DMI as described by Tosto et al. [30] in goats. Our previous findings [18] showed increased energy balance in pre-parturition goats associated with increasing BBR supplementation. Hence, improved milk production in BBR supplemented goats confirms the enhancement of energy balance. The present finding related to the improvement in DMI at kidding and thereafter in does receiving BBR [2 and 4 g/d] together with increased in milk production and BCS, demonstrated that it could be a useful tool to ameliorate metabolic related stress during late gestation and early lactation.

### Glucose metabolism and related hormones

The reduction in glucose concentration during the gestation is typical for goats and ewes as a consequence of rapid fetal growth and gradual reduction in DMI [1, 39]. The sharp increase in plasma glucose at kidding and thereafter is a normal response to parturition-induced endocrine changes that stimulate gluconeogenesis and lipolysis [11, 39]. The temporal changes of plasma glucose and insulin in this study were consistent with previous studies [1, 29].

Glucose and insulin concentration peaked at kidding which may be related to the release of glucocorticoids immediately before kidding that stimulate glycogenolysis and gluconeogenesis [40]. The increase in glucose concentration on days 7 and 14 PP in BBR-supplemented goats compared with CTRL ensured an adequate glucose supply to the mammary gland for milk production, which is also associated with increased milk production in these groups. Similarly, Bach et al. [41] reported that the ability to proportionally direct more of the absorbed nutrients toward milk synthesis is one of the most critical mechanisms determining improved milk yield. Although we are faced with a lack of results regarding the effect of BBR in ruminants, but clinically BBR was assessed in the metabolic syndrome, obesity, T2DM and IR. In patients with type 2 diabetes mellitus, BBR significantly improved the level of fasting blood glucose, the level of postprandial blood glucose and decreased IR [42]. It has been also reported that oral administration of BBR (50 and 100 mg/kg) exerts a beneficial effect on glucose and insulin metabolism in rats [22]. High numbers of studies have identified the mechanisms of action of BBR on glucose and energy metabolism. Among the pathways through which BBR modulates cellular processes, AMP-activated protein kinase (AMPK) plays a central role [21]. AMPK is a cellular energy sensor that regulates the energy homeostasis of body. BBR also regulates the expression of metabolic genes, leading to the enhancement in glucose metabolism [43]. Pirillo and Catapano [44] indicated that BBR enhance glucose and energy homeostasis by increasing expression and activity of glucose transporters 1 and 4 (GLUT1 and GLUT4) via the activation of AMPK and ERK pathways.

Cortisol is an important hormone involved in regulation of either gluconeogenesis or carbohydrate and lipid metabolism [45]. Cortisol acts as a gluconeogenic hormone in cattle [45] and evokes an IR state in dairy cows [46]. Hence, the higher blood cortisol levels, which were found in non-BBR goats reflected severe NEB during late gestation and early lactation. A tendency for decreases in plasma cortisol was observed with increasing BBR supplementation which is in line with our previous results [18] where significant higher EB was found in BBR-treated goats. Similar results were reported by Moyes et al. [47], who reported that NEB led to reduction in blood cortisol levels in dairy cows during early lactation. However, Zamuner et al. [1] pointed out that the comparisons between goats and cows should be done with caution, doe to the different metabolic status. Plasma glucagon concentration was not affected by BBR ingestion. However, the progressive increase in glucagon concentration in early lactation has been previously reported as a homeorhetic adaptation [48]. Overall, the findings of endocrine changes of BBR-goats compared to non-BBR during the transition and early lactation periods supported the concept of improved glucose and insulin metabolism by BBR supplementation.

### Somatotropic Axis in blood plasma

The somatotropic axis mediates essential signals for the successful implementation and maintenance of lactation. GH and IGF-1 contribute markedly to this process during early lactation [49, 50]. The increased GH secretion during the NEB in early PP enables the shift of nutrients from body reserves towards the mammary gland for milk synthesis [49] with a subsequent uncoupling of GH-IGF-1 [49, 50]. The current observed changes in plasma GH and IGF-1 concentration of goats around kidding and early lactation agree with the results of Hashizume et al. [11] who reported a progressive increase in plasma GH as kidding approached as well as a gradual reduction in plasma IGF-I in late gestation until the day of parturition. The changes in blood plasma GH and IGF-I around the late gestation and early lactation related to the changes in the goat’s energy balance [1, 11] as well as in cows [34, 50]. It is well established that NEB is related to increased plasma GH and decreased plasma IGF-I concentrations [50]. Data from this study indicated that plasma GH was lower in BBR treated groups than non-BBR goats. Likewise, BBR supplementation increased plasma IGF-I concentration in dairy goats during early lactation. In agreement with our companion paper [18], described a positive effect of BBR supplementation on energy balance in goats. Generally, an inadequate energy status is related to an uncoupling of the somatotropic axis, resulting in an increased of GH and a decreased in IGF-1 concentrations in blood [7, 8]. Because the liver significantly contributes to the systemic somatotropic axis, the NEB during the transition period leads to changes in key factors in the somatotropic axis in the liver. Earlier studies in dairy goats revealed that higher concentrations of IGF-I might play an important role in supporting metabolism during the last stages of pregnancy and early postpartum [11]. It is also assumed that the modulation of the somatotropic axis (i.e., elevated IGF-I by decreased GH) takes place when glucose and insulin concentrations in blood plasma are elevated in dairy cows during the transition period [7, 50].

In addition, plasma concentration of IGF-I increased with increasing BBR dosage in goats. In this regard, BBR has been suggested as a modulator of somatotropic axis [51, 52]. BBR (25 and 50 mg/kg) significantly increased mRNA expression of IGF-1 in sciatic nerve of rat [52]. It also has been reported that BBR activates multiple cellular pathways including PPAR-g and AMPK, which associated in somatotropic axis modulation by enhancing IGF-1 [16, 52]. Interestingly, Yu et al. [21] reported that BBR can support pancreatic b-cell proliferation and stimulate insulin secretion in Min6 islet b-cell lines as well as activation of cell regulatory proteins (ERK1/2) so that the insulin receptor substrate IRS-2 expression increases activation of the insulin/insulin-like growth factor (IGF-1) signaling cascades. In line with our findings, a recent paper [53] showed that the supplementation of fish with barberry extract (a natural compound rich in BBR) led to higher IGF-I concentrations. In sum, the improved energy status in BBR-treated goats was associated with an improved glucose and insulin status, the stimulation of the somatotropic axis in the present study was closely related to enhanced glucose and insulin availability in these goats [11, 54]. It should be noted that due to the lack of information on dairy goats, it is rather difficult to compare our results with those reported in the literature. Further research is needed to determine the exact mechanism of BBR on the somatotropic axis modulation in dairy goats.

### Glucose and insulin response during IVGTT

It is accepted that changes in responsiveness to insulin during the transition from late gestation to early lactation is a natural homeorhetic adaptation that occurs in goats [1] similar to dairy cows [5]. This promotes glucose availability for non-insulin-responsive glucose uptake by other tissues, including growing fetus and mammary gland [5]. The IVGTT is frequently used to assess systemic glucose metabolism and insulin sensitivity in transition dairy cow and goats [1, 5, 28]. According to De Koster and Opsomer [5], based on data from an IVGTT, insulin resistance identified by lesser glucose and insulin CR, greater AUC, and higher the T_1/2_ and T_base_. In the current study, higher insulin AUC in CTRL goats means that they had to release more insulin in order to remove the same amount of glucose from blood [5]. Although lesser insulin AUC in BBR-supplemented goats is associated with greater glucose tolerance. Insulin resistance is due to either a loss in tissue responsiveness when the maximal response to insulin is reduced or a loss in insulin sensitivity when more insulin is needed to elicit similar responses [55]. After glucose injection, the peak of plasma glucose concentration reflects the insulin response and the glucose CR indicates insulin sensitivity [5, 28, 55]. Therefore, in the present study, higher glucose levels at peak, lower CR as well as higher glucose T_1/2_ in the CTRL group indicate IR in dairy goats in early lactation, which in line with earlier reports in dairy goats [1]. Whereas BBR supplementation, decreased the peak of plasma glucose levels and enhanced its CR, indicating an improved insulin response in BBR-treated groups. In addition, it also has been reported that the elevated plasma fatty acid concentrations because of inadequate EB in early lactation could also have suppressed insulin secretion, as fatty acids may adversely influence pancreatic β-cell functions and exacerbates IR [34]. Our previous findings [18] suggested that BBR may alleviate NEB as reflected by lower NEFA circulation. Thus, the greater glucose CR and insulin CR as well as a decline in glucose AUC in goats supplemented with BBR indicated that these goats had facilitated insulin action. On the other hand, more insulin responsiveness in BBR-fed goats may be partially attributed to improved energy balance and lower body fat mobilization [28].

Numerous in vivo studies using rodent models and clinical research with diabetic patients have shown the positive effect of BBR on enhancing insulin sensitivity [33, 56]. Kung et al. [57] showed that berberine increases the expression of insulin receptor substrates (IRS) gene, which acts as a binding protein for transmitting insulin signals. Lou et al. [58] showed that berberine decreased IR by increasing IRS expression in rodent hepatocytes. A study using diabetic rats showed that BBR supplementation stimulated IRS promoters and increased insulin sensitivity by increasing the expression of IRS gene [59]. Interestingly, BBR also activates AMPK, which has been shown to play a role in reducing IR by recent reports [60]. Consistent with our findings, Shi et al. [35] have been shown that BBR play a beneficial role in insulin signaling and mitochondrial respiratory chain function in bovine hepatocytes. However, it is important to note that glucose and insulin metabolism may vary depending on animal species, genotype, and other interspecies factors. Hence, it is only logical to suppose that such positive effects of BBR in monogastric animals would yield similar results in dairy goats, but caution must be paid when extrapolating our data to monogastric animal models. Taken together, based on our data, the glucose and insulin response to IVGTT suggest that insulin sensitivity might have been increased in goats receiving supplemental BBR, possibly by improved glucose and insulin metabolism as well as reducing body reserve mobilization.

## Conclusion

This study determined the impact of BBR supplementation on endocrine alteration and glucose tolerance in dairy goats during the transition from late gestation into the early lactation. Supplementation with BBR2 and BBR4 affected endocrine status of transition dairy goats, which led to enhanced glucose and insulin concentration and tendency to decrease cortical. On days 7 and 14 PP period, supplemental BBR2 and BBR4 alleviated plasma GH concentration. Furthermore, plasma IGF-1 was also enhanced with BBR2 and BBR4 at kidding and during PP. Intravenous glucose tolerance test greater glucose and insulin CR as well as lesser glucose and insulin AUC underlined improved insulin response with BBR ingestion. Together, our results indicated an improved glucose and insulin status along with the modulation of the somatotropic axis and glucose and insulin response to IVGTT in dairy goats supplemented with BBR. Such observation provided a possible explanation for improved energetic status in BBR treated goats which provide a potential novel alterative strategy for the prevention of metabolic dysfunction in transition dairy goats. However, more research is need focused on gene expression to complement out findings on mechanisms of BBR on nutrient partitioning.

## Data Availability

All data generated or analyzed during this study are included in this published article.
